# Acute Myeloid Leukemia Masquerading As Testicular Mass: A Case Report

**DOI:** 10.7759/cureus.47715

**Published:** 2023-10-26

**Authors:** Dinesh Ravikumar, Karthik Ambalavana, Senthil Kumar Elumalai, Niranjan Vijayaraghavan, Navaneetha Lakshmi Ramesh

**Affiliations:** 1 Medical Oncology, Madras Medical College, Chennai, IND; 2 Radiation Oncology, Cancer Institute, Women India Association (WIA), Chennai, IND

**Keywords:** immunohistochemistry, testicular, bone marrow, acute myeloid lekemia, myeloid sarcoma

## Abstract

Myeloid sarcoma (MS) is the occurrence of primitive granulocytic precursors in various extramedullary sites. It can occur as an isolated disease, present concomitantly, or during the relapse of various myeloid neoplasms. A high index of clinical suspicion is warranted owing to its varied clinical presentation, rarity of diagnosis, inadequate immunohistochemical techniques, and challenging treatment. The occurrence of myeloid sarcoma of the testis, either as an independent entity or as an initial presentation of acute myeloid leukemia (AML), is exceedingly uncommon, with only a few documented cases in the literature. In this case study, we present a patient who initially presented with testicular swelling, later identified as MS, and subsequently diagnosed as AML through a bone marrow aspirate. This report discusses the diagnostic difficulties encountered and the available therapeutic options for managing MS.

## Introduction

Myeloid sarcoma (MS) is a neoplasm manifested by an extramedullary proliferation of immature blasts of the myeloid lineage [1]. They are mostly associated with acute myeloid leukemia (AML), especially in cytogenetic abnormalities like t(8; 22), inv(16), and 11q23 [2]. MS is also known as granulocytic sarcomas, chloromas, chloroleukemia, myeloblastomas, and myelocytomas [3-5]. MS commonly involves the head and neck, orbit, lymph nodes, and skin. Rare sites such as the pancreas, breast, and central nervous system have also been reported. MS involvement of the testes is uncommon and is mostly limited to case reports and series [6-8]. We report a case of a patient with no previous medical history who presented with MS of the testes as the first manifestation of AML. Initially, the patient disregarded his symptoms, but he subsequently deteriorated markedly due to extensive lymph nodal and bone marrow involvement. Our intention in reporting this case is to highlight this rare presentation of AML. It can significantly impact patient outcomes if kept in mind and recognized early.

## Case presentation

A 27-year-old male patient presented to the ED with a five-month history of a left testicular mass. Additionally, the patient reported experiencing weight loss, malaise, reduced urine output, and a gradual increase in abdominal pain and distension for three weeks. Initially, the patient had chosen to disregard his symptoms for five months, but he ultimately decided to seek medical assistance when his condition, including abdominal pain, distension, diminished appetite, and weight loss, deteriorated. Upon physical examination, the patient exhibited a distended abdomen with minimal tenderness and an enlarged left testis. Preliminary laboratory analysis revealed biochemical evidence of acute kidney injury, as well as hyperkalemia and hyponatremia. The complete blood count (CBC) displayed mild normocytic anemia and leucocytosis; however, the differential counts and platelet levels were unremarkable. Tumor lysis syndrome was ruled out as the Cairo-Bishop criteria were not met. The levels of serum alpha-fetoprotein, beta human chorionic gonadotropin (beta hCG), and lactate dehydrogenase (LDH) were found to be within the normal range (Table 1).

**Table 1:** Laboratory parameters showing mild anemia, acute kidney injury, hyponatremia, and hyperkalemia at presentation.

Labs	Results	Reference range
Complete blood count		
Hemoglobin (g/dL)	10	12-16
Mean corpuscular volume (fL)	82	80-100
WBCs (10^3^/mcL)	11.52	4.5-11.0
Differential counts	Normal	Normal
Platelets (10^3^/mcL)	160	150-400
Biochemical parameters		
Blood urea (mg/dL)	98	15-40
Serum creatinine (mg/dL)	5.8	0.6-1.2
Serum sodium (mmol/L)	128	135-145
Serum potassium (mmol/L)	5.3	3.5-5.0
Serum tumor markers		
Alpha-fetoprotein (ng/mL)	14	10-20
Beta-human chorionic gonadotropin (mIU/mL)	1	0-5
Lactate dehydrogenase (U/L)	180	140-280

**Figure 1:**
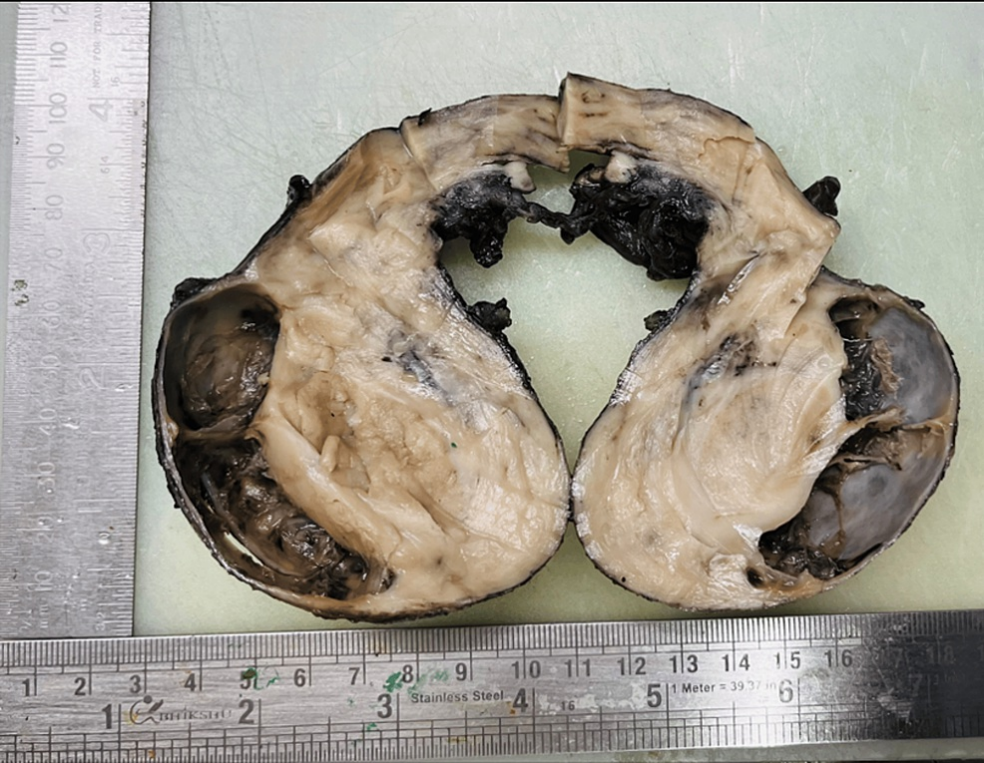
Cross section of testicular mass lesion with areas of necrosis.

**Figure 2:**
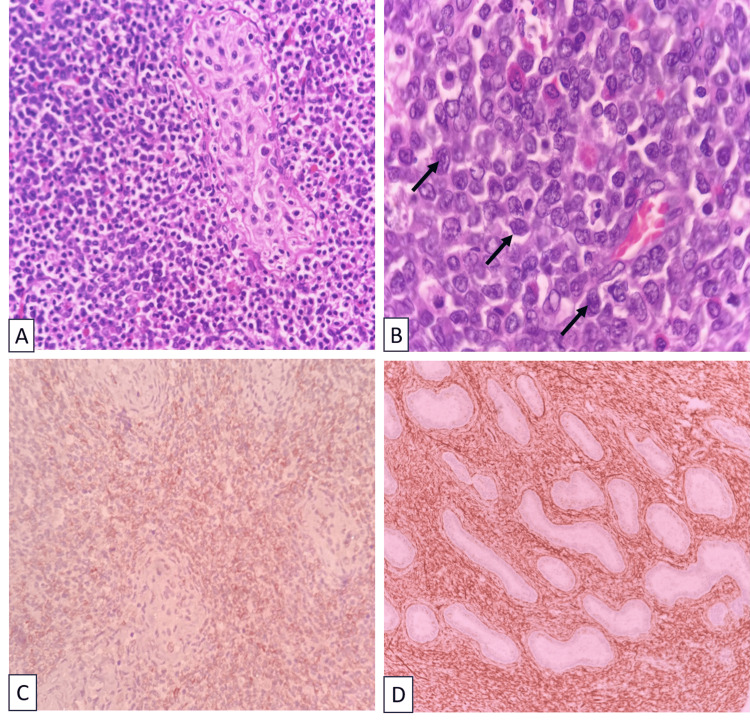
Histopathological examination of the testis Panel A: Testicular parenchyma showing sheets of blasts. Panel B: Sheets of blasts, medium-sized cells with an increased nuclear-cytoplasmic ratio, in a high-power field (H&E, x100). Panel C: CD117 immunopositivity in tumor cells (x100). Panel D: Strong MPO positivity (x100).

CT scans revealed small bowel obstruction secondary to mesenteric lymphadenopathy, bilateral hydroureteronephrosis due to ureteric compression, minimal ascites, and bilateral testicular masses (left>right) with minimal hydrocoele. The patient was managed for acute intestinal obstruction by emergency laparotomy, jejuno-jejunal bypass, mesenteric lymph node biopsy, and postrenal acute kidney injury (AKI), managed with emergency hemodialysis and percutaneous nephrostomy. After recovery from surgery, a high inguinal orchiectomy was done, and samples were sent for histology and immunohistochemistry. On cross-section, the gray-white firm mass measured 7.5x5x3.2 cm (Figure 1). The histology report of the mass shows testicular parenchyma with seminiferous tubules interspersed and surrounded by intermediate- to large-sized neoplastic cells with scant cytoplasm arranged in sheets. Immunohistochemistry demonstrated a strong positive for CD45, CD34, CD117, and myeloperoxidase (MPO) (Figure 2). In contrast, it was negative for ALK-1, Tdt, CD99, CD3, CD1a, CD10, CD19, CD20, Inhibin, and desmin. These results suggest a diagnosis of testicular myeloid sarcoma (TMS).

**Figure 3:**
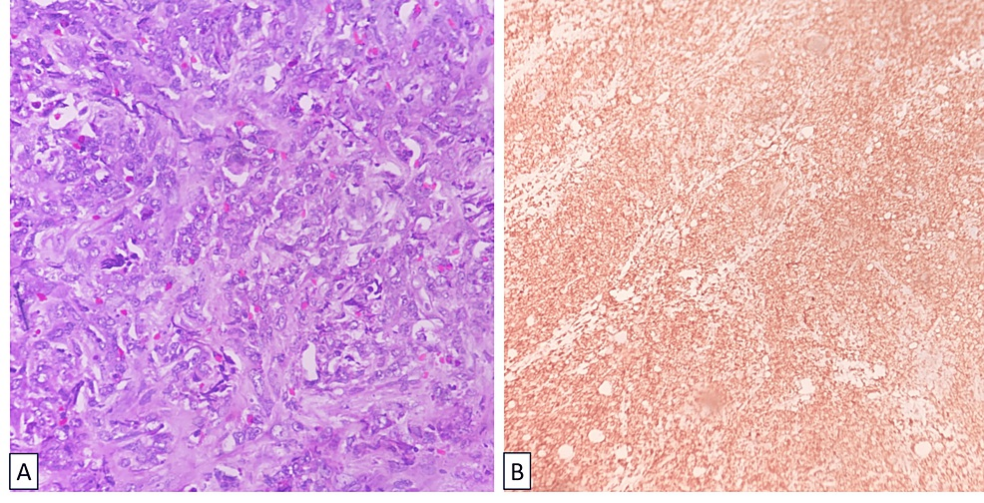
Histopathological examination of mesenteric lymph node. Panel A: Poorly differentiated neoplasm with effacement of underlying architecture by sheets of monomorphic cells. Panel B: Sheets of blasts staining for MPO. MPO: Myeloperoxidase.

A biopsy extracted from the mesenteric lymph node was subjected to histopathological examination. The initial findings indicated the presence of an undifferentiated neoplasm. Subsequently, a diagnosis of myeloid sarcoma was established, parallel to the diagnosis of TMS. The confirmation of the diagnosis was achieved through the detection of MPO positivity on the lymph node (Figure 3). Diagnosing MS on a lymph node posed a challenge due to the absence of any prior history or suspicion of leukemia, which could have guided the pathologists toward suspecting it.

So, a bone marrow aspirate was performed, which confirmed AML with 26% blasts that, from flow cytometry, presented an immunophenotype of CD45+, CD13+, CD33+, CD117+, and MPO. Cytogenetic and molecular analyses were not performed due to low resource settings. The patient attained complete remission of bone marrow and lymphadenopathy after standard induction chemotherapy with daunorubicin and cytarabine (ARA-C). The patient experienced Common Terminology Criteria for Adverse Events (CTCAE) grade 3 febrile neutropenia and grade 2 diarrhea following 3+7 induction chemotherapy. However, blood cultures were unremarkable. The patient was treated with broad-spectrum antibiotics for 10 days and subsequently recovered with additional supportive measures. The patient then underwent consolidation chemotherapy with three cycles of high-dose ARA-C and is currently under evaluation for allogeneic bone marrow transplantation. Due to the patient's initial presentation with acute deterioration of symptoms, the evaluation of anemia was not possible as he was being treated for acute small bowel obstruction. In the ED, a peripheral smear was not conducted due to the absence of any indications or suspicions of hematological malignancy. Moreover, the CBC parameters were largely within normal limits, aside from the presence of mild normocytic anemia and leucocytosis. The diagnosis of AML was further complicated by a prolonged recovery period after surgery and the management of AKI. Subsequently, a bone marrow examination was performed only after the orchiectomy, and the lymph node specimen revealed the presence of MS. 

## Discussion

MS occurs in about 2-8% of AML cases [9]. It is common among young adults, although it can occur at any age. The most common sites affected are bone and soft tissue structures of the head and neck, orbit, ribs, sternum, vertebrae, pelvis, and central nervous system [2]. According to the WHO classification of the tumors of hematopoietic and lymphoid tissues, MS presents as a distinctive tissue-based manifestation of AML, transformed myelodysplastic syndrome (MDS), myeloproliferative neoplasms (MPN) or a combination of MDS and MPN [10]. MS can have varied presentations. (a) Isolated MS: patients without medullary involvement or previous history of malignancies; (b) Secondary MS: patients with a history of AML/MDS/MPN; (c) Concurrent MS: patients with simultaneous diagnosis of TMS and malignancy at the same time [11]. A systematic review conducted by Sahu KK et al. on articles published from 1988 to 2018 revealed that TMS has only been recorded in 68 cases, with only four of those cases being concurrent MS [11]. Table 2 summarises the key studies on concurrent TMS and AML over the last three decades. This case reports that MS of the testis as the presenting sign of AML is uncommon and has a poor prognosis. It also underscores the importance of clinical suspicion by physicians for MS when young patients present with testicular mass. 

**Table 2:** Summary of studies on concurrent testicular myeloid sarcoma and acute myeloid leukemia. B/L: Bilateral; COG-AAML: Children’s Oncology Group (COG) Study AAML; CHOP: Cyclophosphamide, Doxorubicin, Vincristine, and Prednisone; DLBCL: Diffuse Large B Cell Lymphoma; f/b: Followed By; HDUN: Hydroureteronephrosis; HSCT: Hematopoietic Stem Cell Transplantation; IT: Intra Thecal; TMS: Testicular Myeloid Sarcoma.

Case number	Study	Age at the time of diagnosis of TMS, Years	Side	Symptoms	Other sites of MS at the time of diagnosis of TMS	Treatment given
1.	Tran CN et al. [12]	0.5	Left	Testicular pain	None	Induction chemotherapy as per COG-AAML 1031 protocol f/b local radiation therapy and allogeneic HSCT
2.	Wang HQ and Li J [13]	44	Right	Testicular mass	None	Induction (with Idarubicin, cytarabine) f/b consolidation therapy (high-dose cytarabine), IT with cytarabine f/b allogeneic HSCT
3.	Toki H et al. [14]	51	Right	Scrotal mass	Skin nodules, terminal ileum	Initially, CHOP chemotherapy was given for misdiagnosed DLBCL of the terminal ileum. Later, Chemotherapy (Daunorubicin, enocitabine) f/b enocitabine with aclarubicin maintenance chemotherapy was given
4.	Walker BR and Cartwright PC [15]	0.17	Right	Scrotal swelling, skin erythema	None	Chemotherapy (Daunorubicin) and allogeneic HSCT
5.	Our study	27	Left	Testicular mass	Mesenteric lymph nodes causing B/L HDUN	Induction chemotherapy with daunorubicin and cytarabine f/b consolidation with high-dose cytarabine. The patient currently planned for allogeneic HSCT

The accurate histological diagnosis of MS can often be challenging, particularly in cases with no prior history of malignancies. This difficulty also arises due to the diverse clinical presentation of the disease, its rarity, the presence of minimal myeloid differentiation, variable identification of Auer rods in histopathological specimens, and the lack of available literature on the subject [2,11]. Retrospective studies have reported a misdiagnosis rate of approximately 75%, while another series has indicated a misdiagnosis rate ranging from 25% to 47% [16]. Morphological similarities between MS and high-grade non-Hodgkin lymphoma, poorly differentiated carcinomas, plasmacytomas, and other high-grade neoplasms can easily lead to confusion [7,11]. The presence of eosinophilic myelocytes on H&E-stained biopsies may provide a clue to diagnosing MS [17]. Immunohistochemistry is a valuable tool for identifying antigens associated with the myeloid lineage. In this case, immunohistochemistry played a crucial role in narrowing down the diagnosis, as it was positive for CD34, CD117, and MPO, the most expressed markers of myeloid differentiation [11]. A comprehensive analysis of clinical and radiological features, histopathological analysis, appropriate immunohistochemical markers, and molecular profiling are necessary to establish a diagnosis. However, it is essential to maintain a high level of suspicion to achieve an accurate diagnosis [17]. Due to the lack of randomized clinical trials, there is no standardized treatment strategy for MS. Management of TMS usually includes systemic chemotherapy, hematopoietic stem cell transplantation (HSCT), radiation, and surgery [2]. According to the current National Comprehensive Cancer Network guidelines, patients with MS, including testes, should be treated with an AML-type chemotherapeutic regimen [11]. Several AML chemotherapy-like regimens have been used in MS. Tsimberidou AM et al. reported a 69% complete remission rate with ARA-C, idarubicin, and fludarabine in patients with isolated MS [18]. ARA-C-based chemotherapy regimens are mostly used in MS [17]. In our patient, a 3+7 regimen with ARA-C and daunorubicin was used. HSCT is considered the definitive treatment strategy in the management of MS. In retrospective studies, overall survival (OS) benefits had been observed in MS patients who underwent HSCT [9,11]. Radiation is generally used in consolidation after induction chemotherapy, treating localized recurrences after HSCT, and reducing large tumor masses to relieve compression symptoms [2]. Orchidectomy serves both diagnostic and therapeutic purposes [19]. Beyond diagnosis, the surgical procedure can also alleviate symptomatic compression caused by the tumor [2]. In our case, the patient underwent radical orchiectomy, achieved complete remission following induction and consolidation chemotherapy, and is currently undergoing evaluation for allogeneic bone marrow transplantation.

## Conclusions

MS involving the testes is rarely the initial presentation of AML. Our objective in presenting this study is to underscore the importance of keeping MS on the list of differential diagnoses of isolated testicular swelling despite its rarity. Peripheral blood smears are necessary in all cases of testicular swelling. With appropriate use of immunohistochemical techniques, rapid diagnosis can be made, and treatment can be initiated swiftly, regardless of its poor prognosis.
